# The Finnish lapphund retinal atrophy locus maps to the centromeric region of CFA9

**DOI:** 10.1186/1746-6148-3-14

**Published:** 2007-07-10

**Authors:** Jesús Aguirre-Hernández, Kaisa Wickström, David R Sargan

**Affiliations:** 1 Centre for Veterinary Science, University of Cambridge, Madingley Road, Cambridge CB3 0ES, UK; 2 Elainlääkäriasema Akuutti, Kansankatu 47, 90100 Oulu, Finland

## Abstract

**Background:**

Dogs have the second largest number of genetic diseases, after humans. Among the diseases present in dogs, progressive retinal atrophy has been reported in more than a hundred breeds. In some of them, the mutation has been identified and genetic tests have allowed the identification of carriers, thus enabling a drastic reduction in the incidence of the disease. The Finnish lapphund is a dog breed presenting late-onset progressive retinal atrophy for which the disease locus remains unknown.

**Results:**

In this study we mapped the progressive retinal atrophy locus in the Finnish lapphund using a DNA pooling approach, assuming that all affected dogs within the breed share the same identical-by descent-mutation as the cause of the disease (genetic homogeneity). Autosomal recessive inheritance was also assumed, after ruling out, from pedigree analysis, dominant and X-linked inheritance. DNA from 12 Finnish lapphund cases was mixed in one pool, and DNA from 12 first-degree relatives of these cases was mixed to serve as the control pool. The 2 pools were tested with 133 microsatellite markers, 3 of which showed a shift towards homozygosity in the cases. Individual genotyping with these 3 markers confirmed homozygosity for the GALK1 microsatellite only (chromosome 9). Further individual genotyping with additional samples (4 cases and 59 controls) confirmed the association between this marker and the disease locus (p < 0.001). Closely related to this breed are the Swedish lapphund and the Lapponian herder for which a small number of retinal atrophy cases have been reported. Swedish lapphund cases, but not Lapponian herder cases, had the same GALK1 microsatellite genotype as Finnish lapphund cases.

**Conclusion:**

The locus for progressive rod-cone degeneration is known to be close to the GALK1 locus, on the telomeric region of chromosome 9, where the retinal atrophy locus of the Finnish lapphund has been mapped. This suggests that the disease in this breed, as well as in the Swedish lapphund, may correspond to progressive rod-cone degeneration. This would increase the number of known dog breeds having this particular form of progressive retinal atrophy.

## Background

There are more than 350 dog breeds, each maintained as a breeding population separate from other breeds, which are collectively afflicted by more than 450 reported genetic diseases, the incidence of which varies from breed to breed [1,2]. Many of the breeds have been founded from a small number of individuals, and the dogs within them have been subjected to a high degree of inbreeding. In some cases further population bottlenecks and/or popular sire effects have led to a small number of individuals contributing disproportionately to the gene pool of the breed. In small, inbred and genetically isolated populations, inherited diseases are likely to be genetically homogeneous, with the same identical-by-descent mutation underlying all instances of the disease in the breed. Even if there is genetic heterogeneity, one of the mutations may be much more common than the rest due to the aforementioned characteristics of dog breeds. Association studies and, specifically for recessive diseases, autozygosity mapping [3-7], are well suited for mapping disease loci in this kind of populations. Moreover, these approaches have the advantage of not needing DNA samples from members of nuclear families covering several generations, a requirement that may be difficult to meet for late age of onset diseases since by the time a dog is diagnosed as affected, the parents may no longer be alive or sibs and descendants may have been dispersed. In place of nuclear families, association studies may use any affected dogs and unrelated controls. Thus, this approach has the potential for increasing the number of disease loci that may be mapped in this species.

Among canine diseases, progressive retinal atrophy (PRA) involves the gradual death of photoreceptors, first rods, leading to night blindness, and then cones, causing complete loss of sight. PRA has been reported in more than a hundred breeds, and the mutations underlying it have been intensively searched for in many of them, although only few of the mutations have been found [8]. Two different mutations have been identified in *PDE6B*, one causing rod-cone dysplasia type 1 (rcd1) in Irish setters [9-11] and another involved in PRA in Sloughis [12]. Mutations have also been found in *PDC *in the Miniature schnauzer [13], *RPE65 *in Briards with retinal dystrophy [14-16], *PDE6A *in Cardigan Welsh corgis with rod-cone dysplasia type 3 (rcd3) [17], *RHO *in English and Bull mastiffs with autosomal dominant PRA [18], and *RPGR *in Samoyeds and Siberian huskies with X-linked PRA [19]. In addition to this, 3 loci have been mapped: early retinal degeneration (*erd*) in the Norwegian elkhound [20] to CFA27, rod cone dysplasia type 2 (*rcd2*) in the collie [21] to CFA7, and progressive rod-cone degeneration (*prcd*) in CFA9. This last one is the most widespread of these diseases since it occurs in the American and English cocker spaniels, Labrador retriever and Miniature poodle [22]. Crossing experiments involving affected miniature poodles and English and American cocker spaniels showed that *prcd *is allelic in these breeds [23]. The mapping of the PRA locus in the American Eskimo dog suggests the disease in this breed is also *prcd *[7]. Most recently a mutation has been described in a new gene *prcd *which is believed to be causal for this disease [24].

The Finnish lapphund has a long history, descending from Scandinavian spitz type dogs. For a long time it has been a working dog, although its role as a family dog is growing, as its popularity increases, particularly in Finland. The breed standard was first established in 1945, and has been revised several times. Dogs from unregistered parents may still be admitted to the breed if they meet the breed's standard. A number of PRA cases have been found in the breed, but no clinical or genetic studies have been performed so we decided to investigate its cause using genomic mapping techniques.

## Results

### Progressive retinal atrophy in the Finnish lapphund

Affected Finnish lapphunds have been reported in Finland and in the UK; the prevalence of the disease is unknown. By ophthalmoscopic examination it was seen that the early signs of the disease include subtle retinal vascular attenuation, tapetal hyperreflectivity and pale grey optic discs. As the disease progressed tapetal hyperreflectivity and vascular attenuation became more obvious. In some cases there was non-tapetal pigment migration. In dogs ophthalmoscopically examined regularly from birth, the first signs of the disease were noticed at the age of 4 to 6 years, placing it within the late onset group of PRA diseases.

**Figure 1:**
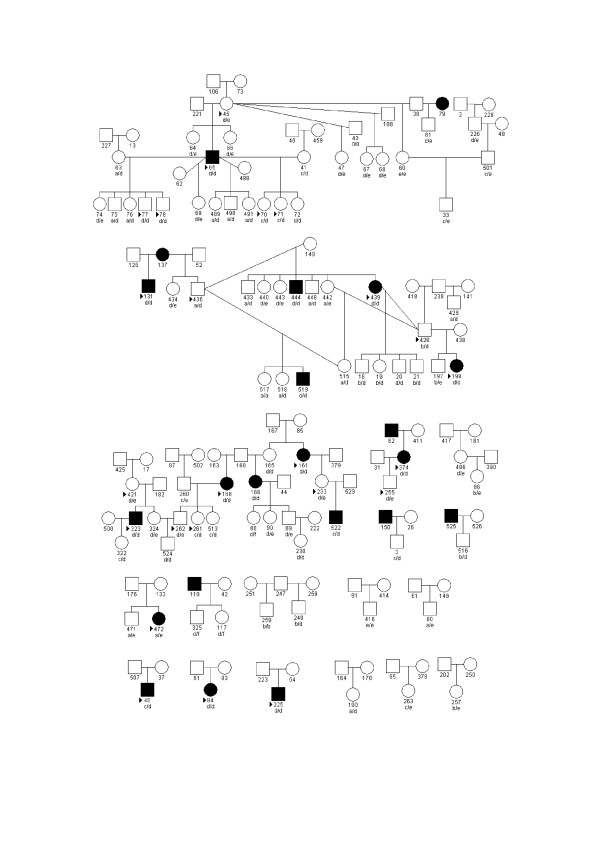
**GALK1 microsatellite genotypes in Finnish lapphunds**. All the individuals included in the study are represented in this figure along with their GALK1 genotype. The allele associated with the disease is "d". Only individuals that have already developed the disease are represented as filled symbols. Arrowheads point to individuals used for DNA pooling.

To date, no multigenerational families have been followed to determine the mode of inheritance of the disease in the Finnish lapphund; however, the distribution of known cases within the breed does not support autosomal dominant or X-linked inheritance (Figure 1).

Pedigree analysis of 21 affected individuals revealed that 16 of them share as a common ancestor one of the founders of the registered breed. That this ancestor is a founder of the registered breed and is itself an obligate carrier suggests that the mutation antedates the registered breed.

### DNA pools and pedigree analysis of the cases

Autozygosity mapping, by DNA pooling was used as the initial step to map the Finnish lapphund PRA locus. To test the suitability of the DNA pooling approach, and to ensure that rare alleles would be detected, thus avoiding false negative results, DNA from two individuals were mixed in different proportions raging from 1:0 to 1:10. These two individuals were heterozygous for the FH2309 marker, having only one allele in common. All three alleles were detected when the DNA were mixed, irrespective of the ratio between the two DNAs. Next, two DNA pools were made, one with DNA from 12 Finnish lapphund cases, and the other with DNA from 12 obligate carriers (parents or offspring of the affected dogs represented in the case pool). The pedigree of the dogs in the case pool was analysed using PedHunter [25] in search of a possible common ancestor. Such a dog was identified as one of the male founders of the registered breed. The number of meioses between the cases and the common ancestor ranged from 8 to 15 (average 11.67). The shortest region of identity by descent [26] was estimated to span 10.05 cM and corresponded to the affected dog furthest removed (at 15 meioses) from the common ancestor.

### Locus mapping

To map the locus, 133 microsatellite markers were studied. Seventeen of these were chosen for their proximity to 14 loci known to be involved in dog or human retinal diseases, or being expressed in the retina: *ABCA4*, *CNGA1*, *GNAT1*, *GNGT1*, *NRL*, *PDC*, *PDE6A*, *PDE6D*, *PDEG*, *PRCD*, *RDS*, *RLBP1*, *ROM1 *and *SAG*. The rest of the markers were selected to cover the whole of the autosomes at intervals of approximately 20 cM. For 117 markers (87.97%) similar levels of heterozygosity were observed in both pools; 13 markers (9.77%) were uninformative, and 3 markers (2.26%) exhibited a shift towards homozygosity in the case pool: FH2175 (CFA16), FH2189 (CFA14) and GALK1 (CFA9).

Individual genotyping was performed with the 3 markers exhibiting the shift, using the same 12 cases and 12 controls used in the DNA pools. Only for GALK1 was the shift towards homozygosity confirmed, with 11 cases homozygous for the same allele, and the remaining one heterozygous. In contrast to this, half of the controls were heterozygous (all of them having one copy of the same allele as the cases, as expected from obligate carriers), and the rest were homozygous for the same allele as the cases. The CLUMP test [27] gave some support to the association between GALK1 and the locus for the disease in this small set of samples (T1 p = 0.10465, T2 p = 0.024138, T3 p = 0.024184, T4 p = 0.060339).

To characterise in more detail the region close to GALK1, additional markers were added and tested with the DNA pools. The markers and their position in the dog genome [28] (version 2.1) were: TK1 (7.5 Mb), FH3359 (10.34 Mb), C03304 (14.28 Mb), FH2263 (16.42 Mb), REN198P23 (18.09 Mb), FH3596 (18.12 Mb), and C09.173 (18.86 Mb). However, no differences in the level of heterozygosity were observed between the pools and no homozygosity was observed among the cases.

### GALK1 individual genotyping

Next, 4 cases and 59 controls were added to the previous set, and all 87 samples were tested for the GALK1 marker using a fluorescent-labelled primer (Fig. 1). The allele frequencies among cases and controls were significantly different (T1 p = 0.001164, LOD = 2.93; T2 p = 0.000046, LOD = 4.34; T3 p = 0.000011, LOD = 4.96; T4 p = 0.000455, LOD = 3.34). T3 assumes association of the mutation with a single allele and the LOD approaches 5.

### GALK1 analysis in Swedish lapphunds and Lapponian herders

A small number of retinal atrophy cases have also been reported for the Swedish lapphund, which is a breed closely related to the Finnish lapphund, so a group of 7 samples was assembled. The 3 cases belonged to a nuclear family (2 full sibs and a half sib to them, with ages at diagnosis of 12 years 7 months and 11 years 5 months for the full sibs, and 9 years 4 months for the half sib). Among the controls, 3 belonged to a different nuclear family (2 half-sibs and a maternal uncle), while a fourth control belonged to a separate family.

All 3 cases in the Swedish lapphund were homozygous for the same GALK1 microsatellite allele (allele "d") as affected Finnish lapphunds, while all controls were heterozygous and all of them carried one copy of allele d.

The Lapponian herder is a breed closely related to the two previous ones and a few retinal atrophy cases have also been reported. Clinically, the disease appears to be similar to the one observed in the Finnish lapphund.

Samples from 3 Lapponian herder cases and 6 controls (including four obligate carriers) were obtained. The 2 cases from Finland were diagnosed at 3 years 10 months and 3 years 1 month. The third case, from Norway, was diagnosed at 3 years 3 months. All 3 cases were heterozygous for the GALK1 marker, and only one of the 6 alleles in this group corresponded to allele d, the one present in homozygous state among the cases in the other two breeds. In the controls, only an obligate carrier dam had one copy of allele d but it was not transmitted to a descendant case.

## Discussion

We have used DNA pooling [29-32], a particular form of autozygosity mapping, to map the PRA locus in the Finnish lapphund, using a low marker density and a small initial set of cases and controls. The results suggest that the PRA disease in the Finnish lapphund may be *prcd*. The study that mapped the *prcd *locus established its location in the centromeric region of chromosome 9 and all cases in that study had the same homozygous genotype for the GALK1 microsatellite [22,33]. Homozygosity mapping of the PRA locus in the American Eskimo dog also showed that all affected dogs were homozygous for the GALK1 marker, while six different alleles were seen among the controls [7]. As in other breeds with *prcd*, the disease in the Finnish lapphund is a late-onset retinal degeneration with signs progressing from mild to severe. However, no electroretinogram or ultrastructural studies have been performed in this breed.

The initial results with pooled DNA showed a reduction in heterozygosity for the GALK1 marker in the cases, when compared to the controls. Individual genotyping of the individuals represented in the DNA pools confirmed the homozygosity for all the cases, except one, while among the controls heterozygous genotypes were observed, as expected from obligate carriers, plus some homozygous individuals. The single heterozygous genotype observed among the cases in the DNA pooling stage may be due to a recombination event. Alternatively, it may represent a phenocopy, having a retinal disease different from that in the rest of the cases. If this case has the same disease as the rest of affected Finnish lapphunds, excluding the GALK1 region on the basis of this single heterozygote may lead to a false negative result. A certain number of non-homozygous cases are expected in the region harbouring the disease locus, due to recombination events [26]. The proportion of these individuals depends both on the number of meioses between the origin of the mutation and the cases being examined, and on the average spacing between the markers used [26]. The CLUMP tests applied to the individual genotyping results of these 12 cases and 12 controls resulted in significant differences for tests T2 and T3. Tests T1 and T4 were non-significant. This differences in the level of significance are the result of the different ways in which the tests handle the alleles, starting from the same contingency test. The levels of significance would also have been influenced by the small number of samples used (24 alleles in each set) and by the fact that the controls were all obligate carriers, meaning that allele differences between the two sets would be smaller than that if non-related controls had been used. At least half of the alleles in the initial controls were expected to be shared with the cases and, as mentioned before, some controls had the same genotype as the cases. Taking all of this into account, it was considered that PRA locus for this breed could be close to the GALK1 marker. When additional samples were tested, all four tests were significant, with T2 and T3 again showing the highest values.

Other markers on chromosome 9 did not show a shift towards homozygosity in the DNA pools. Given that the results for the GALK1 marker were significant, and that no other markers showed a shift towards homozygosity that could be corroborated with individual genotyping, it is considered unlikely that the GALK1 region may represent a false positive.

The individual genotyping of a larger set of Finnish lapphunds resulted in three cases, out of a total of 16, heterozygous for the GALK1 microsatellite. Two of them (individuals 522 and 40 in Figure 1) had one copy of allele d, which was homozygous for the other cases, while this allele was absent from the other case (individual 472). In the third case, which does not share any allele with the rest of the cases, recombination events may have occurred in the lineage leading to it. Alternatively, this individual may have a different retinal disease. This dog was diagnosed at the age of 4 years 8 months and its characteristics were similar to those of other cases in this breed: slight hyper reflectivity, thin veins and pale grey optic nerve head. The dog was examined three times (at ages of 1 year 8 months, 2 years 8 months and 3 years 8 months) before the first signs of disease appeared. The last examination was done at 5 years and 6 months. There was very little or no progression and no pigment migration. It is possible that a different retinal disease is present in this individual. Finding the PRA mutation in the Finnish lapphund may help to solve this case.

Some of the individuals used as controls were very young so it is possible that some may develop the disease in the future, as they grow older. According to the results of the GALK1 microsatellite, some of them share the same genotype as the cases. In association studies, as in any other mapping approach, phenotypes of cases and controls should be correctly assigned. However, this may be difficult sometimes. For example, in late-onset diseases there is the risk of including as controls some individuals which may not have yet developed the disease. Nevertheless, even when a small number of individuals are incorrectly assigned to one set or another, it may still be possible to map the disease locus [34], although with some loss of power. In this context, it has been suggested that unscreened individuals, being plentiful and easy to obtain, may be used as controls, in situations where screening them is difficult or impossible, as long as enough controls are included in the study to compensate for the loss of power derived from the inclusion of some cases among the controls [35].

In the Swedish lapphund and the Lapponian herder, which are breeds closely related to the Finnish lapphund, some PRA cases have also been reported. This suggests they may all have the same type of PRA. For the GALK1 microsatellite, the genotype of affected Swedish lapphunds was the same as in Finnish lapphunds. However, for the Lapponian herder only one of the three cases had the same allele as the one present in the PRA cases of the other 2 breeds. This could imply that there have been recombination events between GALK1 and the PRA locus in this breed, or that the retinal disease is a different one. Studying a larger set of individuals, or identifying the mutation in the other 2 breeds, may help solve this problem.

## Conclusion

Association studies, including autozygosity mapping may be a practical way of mapping genes underlying canine diseases, using dogs from the pet population, since this approach exploits the particular characteristics of dog breed populations. The PRA locus in the Finnish lapphund was mapped to the centromeric region of choromosome 9, which harbours the *prcd *locus. If the gene underlying the disease in the Scandinavian breeds is confirmed to be the same as that causing *prcd*, this would add 2 more breeds to those already known to have this form of PRA.

## Methods

### Samples

Blood samples and pedigree information were obtained from breeders, pet owners and veterinarians. The status of Finnish lapphunds from Finland was determined by ophthalmoscopic eye examination by one of us (KW). Dogs were examined by indirect ophthalmoscopy and slit lamp biomicroscopy under the Finnish Kennel Club program for eradication of inherited eye diseases in dogs. Mydriasis was induced in all eyes with topical tropicamide. First degree relatives of Finnish lapphund cases were called for eye-examination. Some individuals from this breed, as well as Swedish lapphunds and Lapponian herders, were unavailable for ophthalmoscopic examination by the authors, so the status of the dogs was determined from eye-test certificates obtained from national eye schemes administered through veterinary ophthalmic specialists. The average age for the controls was 7 years 4 months (standard deviation 2.7, 95% confidence interval 0.022).

### Pedigree analysis

PedHunter [25] was used to search for the most recent common ancestor of the cases' parents and to determine the number of meioses from the cases to this ancestor. The length of the smallest region identical by descent [26] was estimated from the number of meioses separating the cases from the common ancestor, assuming a length of 26.5 Morgans for the canine genome [36].

### DNA pooling

For the DNA pooling experiments, DNA from 12 cases (the "case pool") was pooled by mixing equal amounts of DNA from each one of them. For the controls ("control pool") equal amounts of DNA were mixed from 12 first degree relatives (parents or offspring) of the cases represented in the case pool; thus, all these relatives were obligate carriers. Seventeen of the 133 microsatellite were less than 12 Mb from genes known to be involved in retinal diseases in dogs or humans, or known to be expressed in the retina (*ABCA4*, *CNGA1*, *GNAT1*, *GNGT1*, *NRL*, *PDC*, *PDE6A*, *PDE6D*, *PDEG*, *PRCD*, *RDS*, *RLBP1*, *ROM1 *and *SAG*). The rest of the markers were distributed throughout the dog genome. The average spacing between the 133 markers was 20 cM. Markers were amplified, using the pooled DNA, with radiolabelled forward primers ([γ-^32^P]ATP, Amersham Biosciences, 10 μCi/μl, 3000 Ci/mmole) and touchdown-PCR. Samples were run in denaturing sequencing gels and detected by autoradiography. For the markers showing a shift towards homozygosity in the case pool, the results were confirmed through individual genotyping of the original set of 12 cases and 12 controls using the same procedure as above.

After this initial mapping showed the presence of the locus on chromosome 9, additional markers on this chromosome were studied with the DNA pools as described above, as well as by individual genotyping.

### GALK1 individual genotyping

Further individual genotyping for the GALK1 microsatellite marker was done in a larger set of Finnish lapphunds, as well as in a group of Swedish lapphunds and Lapponian herders, with a dye-labelled forward primer and a CEQ 8000 automatic sequencer (Beckman Coulter).

### Association analysis

Finnish lapphund GALK1 microsatellite allele distribution in cases and controls was analysed with the CLUMP program [27], version 23. This program calculates the chi squared value of the observed results and assesses its significance by determining the number of times that value is obtained from randomly generated data in contingency tables with the same marginal values as those in the table with the observed results. Four tests are performed with this program. T1 is the chi square obtained from the original contingency table. For the rest of the tests (T2, T3 and T4) the columns of the original table are rearranged before estimating the chi squared value; the level of significance is determined from simulated data in tables with the same marginal values as the rearranged tables. For T2, alleles with expected values of less than 5 are lumped together; for T3 a 2-by-2 table is generated from the observed values by comparing each column in turn against the rest of the clumped columns; the table giving the maximum chi squared value is used to simulate tables with the same marginal totals. For T4, a 2-by-2 table is obtained from the original table by clumping columns so that the maximum chi-squared value is obtained. LOD scores were calculated from the CLUMP output p values as log(1-p/p), where p is the probability that there is no difference between the observed allele distribution (in cases and controls) and that expected based on the distribution in the whole population.

## Authors' contributions

JAH determined the experimental approach, performed the experiments, analysed the results and wrote the first draft of the manuscript. KW identified the cases and their relatives, performed the clinical studies, collected the samples, participated in the analysis of the results and took part in writing the manuscript. DRS conceived and coordinated the project, obtained funding, analysed the results and took part in writing the manuscript.

## References

[B1] Sargan DR (2004). IDID: inherited diseases in dogs: web-based information for canine inherited disease genetics. Mamm Genome.

[B2] Sutter NB, Ostrander EA (2004). Dog star rising: the canine genetic system. Nat Rev Genet.

[B3] Lander ES, Botstein D (1987). Homozygosity mapping: a way to map human recessive traits with the DNA of inbred children. Science.

[B4] Mueller RF, Bishop DT (1993). Autozygosity mapping, complex consanguinity, and autosomal recessive disorders. J Med Genet.

[B5] Farrall M (1993). Homozygosity mapping: familiarity breeds debility. Nat Genet.

[B6] Sheffield VC, Stone EM, Carmi R (1998). Use of isolated inbred human populations for identification of disease genes. Trends Genet.

[B7] Moody JA, Famula TR, Sampson RC, Murphy KE (2005). Identification of microsatellite markers linked to progressive retinal atrophy in American Eskimo Dogs. Am J Vet Res.

[B8] Aguirre-Hernandez J, Sargan DR (2005). Evaluation of candidate genes in the absence of positional information: a poor bet on a blind dog!. J Hered.

[B9] Clements PJ, Gregory CY, Peterson-Jones SM, Sargan DR, Bhattacharya SS (1993). Confirmation of the rod cGMP phosphodiesterase beta subunit (PDE beta) nonsense mutation in affected rcd-1 Irish setters in the UK and development of a diagnostic test. Curr Eye Res.

[B10] Ray K, Baldwin VJ, Acland GM, Blanton SH, Aguirre GD (1994). Cosegregation of codon 807 mutation of the canine rod cGMP phosphodiesterase beta gene and rcd1. Invest Ophthalmol Vis Sci.

[B11] Suber ML, Pittler SJ, Qin N, Wright GC, Holcombe V, Lee RH, Craft CM, Lolley RN, Baehr W, Hurwitz RL (1993). Irish setter dogs affected with rod/cone dysplasia contain a nonsense mutation in the rod cGMP phosphodiesterase beta-subunit gene. Proc Natl Acad Sci USA.

[B12] Dekomien G, Runte M, Godde R, Epplen JT (2000). Generalized progressive retinal atrophy of Sloughi dogs is due to an 8-bp insertion in exon 21 of the PDE6B gene. Cytogenet Cell Genet.

[B13] Zhang Q, Acland GM, Parshall CJ, Haskell J, Ray K, Aguirre GD (1998). Characterization of canine photoreceptor phosducin cDNA and identification of a sequence variant in dogs with photoreceptor dysplasia. Gene.

[B14] Aguirre GD, Baldwin V, Pearce-Kelling S, Narfstrom K, Ray K, Acland GM (1998). Congenital stationary night blindness in the dog: common mutation in the RPE65 gene indicates founder effect. Mol Vis.

[B15] Veske A, Nilsson SE, Narfstrom K, Gal A (1998). Retinal dystrophy of Swedish briard/briard-Beagle dogs is due to a 4-bp deletion in the canine RPE65 gene. Am J Hum Genet.

[B16] Veske A, Nilsson SE, Narfstrom K, Gal A (1999). Retinal dystrophy of Swedish briard/briard-beagle dogs is due to a 4-bp deletion in RPE65. Genomics.

[B17] Petersen-Jones SM, Entz DD, Sargan DR (1999). cGMP phosphodiesterase-alpha mutation causes progressive retinal atrophy in the Cardigan Welsh corgi dog. Invest Ophthalmol Vis Sci.

[B18] Kijas JW, Cideciyan AV, Aleman TS, Pianta MJ, Pearce-Kelling SE, Miller BJ, Jacobson SG, Aguirre GD, Acland GM (2002). Naturally occurring rhodopsin mutation in the dog causes retinal dysfunction and degeneration mimicking human dominant retinitis pigmentosa. Proc Natl Acad Sci USA.

[B19] Zhang Q, Acland GM, Wu WX, Johnson JL, Pearce-Kelling S, Tulloch B, Vervoort R, Wright AF, Aguirre GD (2002). Different RPGR exon ORF15 mutations in Canids provide insights into photoreceptor cell degeneration. Hum Mol Genet.

[B20] Acland GM, Ray K, Mellersh CS, Langston AA, Rine J, Ostrander EA, Aguirre GD (1999). A novel retinal degeneration locus identified by linkage and comparative mapping of canine early retinal degeneration. Genomics.

[B21] Kukekova AV, Nelson J, Kuchtey RW, Lowe JK, Johnson JL, Ostrander EA, Aguirre GD, Acland GM (2006). Linkage mapping of canine rod cone dysplasia type 2 (rcd2) to CFA7, the canine orthologue of human 1q32. Invest Ophthalmol Vis Sci.

[B22] Acland GM, Ray K, Mellersh CS, Gu W, Langston AA, Rine J, Ostrander EA, Aguirre GD (1998). Linkage analysis and comparative mapping of canine progressive rod-cone degeneration (prcd) establishes potential locus homology with retinitis pigmentosa (RP17) in humans. Proc Natl Acad Sci USA.

[B23] Aguirre GD, Acland GM (1988). Variation in retinal degeneration phenotype inherited at the prcd locus. Exp Eye Res.

[B24] Zangerl B, Goldstein O, Philp AR, Lindauer SJ, Pearce-Kelling SE, Mullins RF, Graphodatsky AS, Ripoll D, Felix JS, Stone EM (2006). Identical mutation in a novel retinal gene causes progressive rod-cone degeneration in dogs and retinitis pigmentosa in humans. Genomics.

[B25] Agarwala R, Biesecker LG, Hopkins KA, Francomano CA, Schaffer AA (1998). Software for constructing and verifying pedigrees within large genealogies and an application to the Old Order Amish of Lancaster County. Genome Res.

[B26] Genin E, Todorov AA, Clerget-Darpoux F (1998). Optimization of genome search strategies for homozygosity mapping: influence of marker spacing on power and threshold criteria for identification of candidate regions. Ann Hum Genet.

[B27] Sham PC, Curtis D (1995). Monte Carlo tests for associations between disease and alleles at highly polymorphic loci. Ann Hum Genet.

[B28] Lindblad-Toh K, Wade CM, Mikkelsen TS, Karlsson EK, Jaffe DB, Kamal M, Clamp M, Chang JL, Kulbokas EJ, Zody MC (2005). Genome sequence, comparative analysis and haplotype structure of the domestic dog. Nature.

[B29] Sheffield VC, Nishimura DY, Stone EM (1995). Novel approaches to linkage mapping. Curr Opin Genet Dev.

[B30] Barcellos LF, Klitz W, Field LL, Tobias R, Bowcock AM, Wilson R, Nelson MP, Nagatomi J, Thomson G (1997). Association mapping of disease loci, by use of a pooled DNA genomic screen. Am J Hum Genet.

[B31] Sheffield VC, Carmi R, Kwitek-Black A, Rokhlina T, Nishimura D, Duyk GM, Elbedour K, Sunden SL, Stone EM (1994). Identification of a Bardet-Biedl syndrome locus on chromosome 3 and evaluation of an efficient approach to homozygosity mapping. Hum Mol Genet.

[B32] Carmi R, Rokhlina T, Kwitek-Black AE, Elbedour K, Nishimura D, Stone EM, Sheffield VC (1995). Use of a DNA pooling srategy to identufy a human obesity syndrome locus on chromosome 15. Human Molecular Genetics.

[B33] Sidjanin DJ, Miller B, Kijas J, McElwee J, Pillardy J, Malek J, Pai G, Feldblyum T, Fraser C, Acland G (2003). Radiation hybrid map, physical map, and low-pass genomic sequence of the canine prcd region on CFA9 and comparative mapping with the syntenic region on human chromosome 17. Genomics.

[B34] Edwards BJ, Haynes C, Levenstien MA, Finch SJ, Gordon D (2005). Power and sample size calculations in the presence of phenotype errors for case/control genetic association studies. BMC Genet.

[B35] Moskvina V, Holmans P, Schmidt KM, Craddock N (2005). Design of case-controls studies with unscreened controls. Ann Hum Genet.

[B36] Neff MW, Broman KW, Mellersh CS, Ray K, Acland GM, Aguirre GD, Ziegle JS, Ostrander EA, Rine J (1999). A second-generation genetic linkage map of the domestic dog, Canis familiaris. Genetics.

